# Pancreatic ductal adenocarcinoma arising from the pancreatic parenchyma compressed by a huge pancreatic lipoma: a case report

**DOI:** 10.1186/s40792-023-01720-w

**Published:** 2023-08-01

**Authors:** Seiji Nakahara, Shunsuke Doi, Satoshi Nishiwada, Satoshi Yasuda, Minako Nagai, Kota Nakamura, Yasuko Matsuo, Taichi Terai, Yuichiro Kohara, Takeshi Sakata, Yuji Nitta, Kohei Morita, Masayuki Sho

**Affiliations:** 1 Department of Surgery, Nara Medical University, 840 Shijo-Cho, Kashihara, Nara 634-8522 Japan; 2 Department of Diagnostic Pathology, Nara Medical University, 840 Shijo-Cho, Kashihara, Nara 634-8522 Japan

**Keywords:** Pancreatic ductal adenocarcinoma, Pancreatic lipoma, Pancreatic liposarcoma, Chronic pancreatitis

## Abstract

**Background:**

Pancreatic lipomas (PLs) arising from the adipose tissue in the pancreatic parenchyma are rare among pancreatic tumors. Coexisting pancreatic ductal adenocarcinoma (PDAC) and PLs have not been previously reported. Herein, we report a case of PDAC arising from the pancreatic parenchyma with chronic pancreatitis compressed by a large PL.

**Case presentation:**

The patient was a 69-year-old male. He had been diagnosed with a PL using computed tomography (CT) 12 years previously. The tumor had been slowly growing and was followed up carefully because of the possibility of well-differentiated liposarcoma. During follow-up, laboratory data revealed liver damage and slightly elevated levels of inflammatory markers. Contrast-enhanced CT revealed the previously diagnosed 12 cm pancreatic head tumor and an irregular isodensity mass at the upper margin of the tumor that invaded and obstructed the distal common bile duct. Magnetic resonance cholangiopancreatography demonstrated no specific findings in the main pancreatic duct. Based on these imaging findings, the patient underwent endoscopic retrograde biliary drainage and bile duct brushing cytology, which revealed indeterminate findings. The differential diagnosis of the tumor at that time was as follows: (1) pancreatic liposarcoma (focal change from well-differentiated to dedifferentiated, not lipoma), (2) distal cholangiocarcinoma, and (3) pancreatic cancer. After the cholangitis improved, a pancreatoduodenectomy was performed. Histologically, hematoxylin–eosin staining revealed moderately differentiated PDAC compressed by proliferating adipose tissue. The adipose lesion showed homogeneous adipose tissue with no evidence of sarcoma, which led to a diagnosis of lipoma. Additionally, extensive fibrosis of the pancreatic parenchyma and atrophy of the acinar cells around the lipoma was suggestive of chronic pancreatitis. The pathological diagnosis was PDAC (pT2N0M0 pStage Ib) with chronic pancreatitis and PL. The postoperative course was uneventful, and the patient was discharged on the 15th day after surgery. The patient received adjuvant chemotherapy and has remained recurrence-free for more than 6 months.

**Conclusions:**

PL may be associated with the development of PDAC in the surrounding inflammatory microenvironment of chronic pancreatitis. In cases of growing lipomas, careful radiologic surveillance may be needed not only for the possibility of liposarcoma but also for the coincidental occurrence of PDAC.

## Introduction

Although lipomas are one of the most common benign mesenchymal tumors, pancreatic lipomas (PLs), arising from adipose tissue in the pancreatic parenchyma, are extremely rare among pancreatic tumors [1]. Previous studies have reported that 0.012–0.08% of consecutive patients undergoing computed tomography (CT) or magnetic resonance imaging (MRI) were incidentally diagnosed with PL; however, the accurate incidence of PL remains unclear [2, 3]. Lipomas must be strictly distinguished from liposarcomas, which are malignant tumors derived from adipose tissues. However, this is sometimes difficult because of radiographic similarities, especially with well-differentiated liposarcomas [4].

Surgical resection of lipomas is considered in cases of suspected liposarcomas with invasion of other organs or the presence of solid components [5]. On the other hand, cases of overlapping tumors between PLs and other pancreatic tumors are extremely rare; only one case of PL with benign intraductal papillary mucinous neoplasm has been reported to date [6]. Furthermore, cases of coexisting pancreatic ductal adenocarcinoma (PDAC) and PL have not been reported. Herein, we report a case of PDAC arising from the pancreatic parenchyma with chronic pancreatitis compressed by a large PL.

## Case presentation

**Fig. 1:**
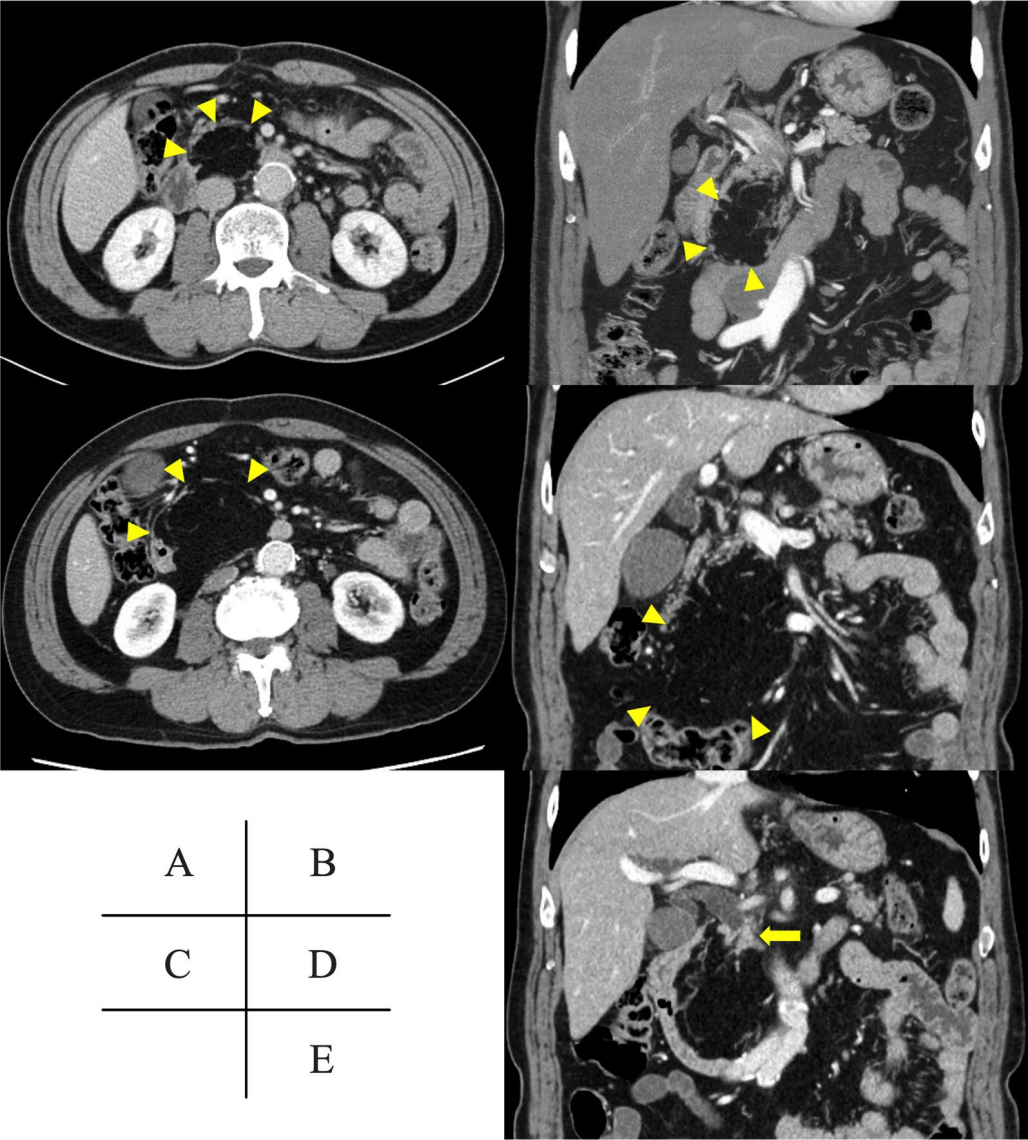
**A, B** Contrast-enhanced CT performed when the patient was diagnosed with pancreatic lipoma 12 years previously. A well-defined 6 cm mass composed of homogeneous adipose tissue without any solid nodules in the pancreatic head is visible (arrowheads). **C–E** Contrast-enhanced CT before surgery. The previously diagnosed 12 cm adipose tumor is visible in the pancreatic head (arrowheads). An irregular isodense mass appeared at the upper margin of the tumor (arrow), which invaded and obstructed the distal common bile duct

The patient was a 69-year-old male. He was referred to our department because of the detection of a pancreatic mass on a screening contrast-enhanced CT screening. Initial contrast-enhanced CT revealed a well-defined 6 cm mass composed of homogeneous adipose tissue without any solid nodules in the pancreatic head, which led to the diagnosis of PL (Fig. 1A, B). In addition to these imaging findings, since he had no symptoms, he was followed up with MRI once a year. Because the tumor had been slowly growing to 12 cm over 11 years, we considered the possibility that it was a well-differentiated liposarcoma and continued to follow the patient closely.

**Fig. 2:**
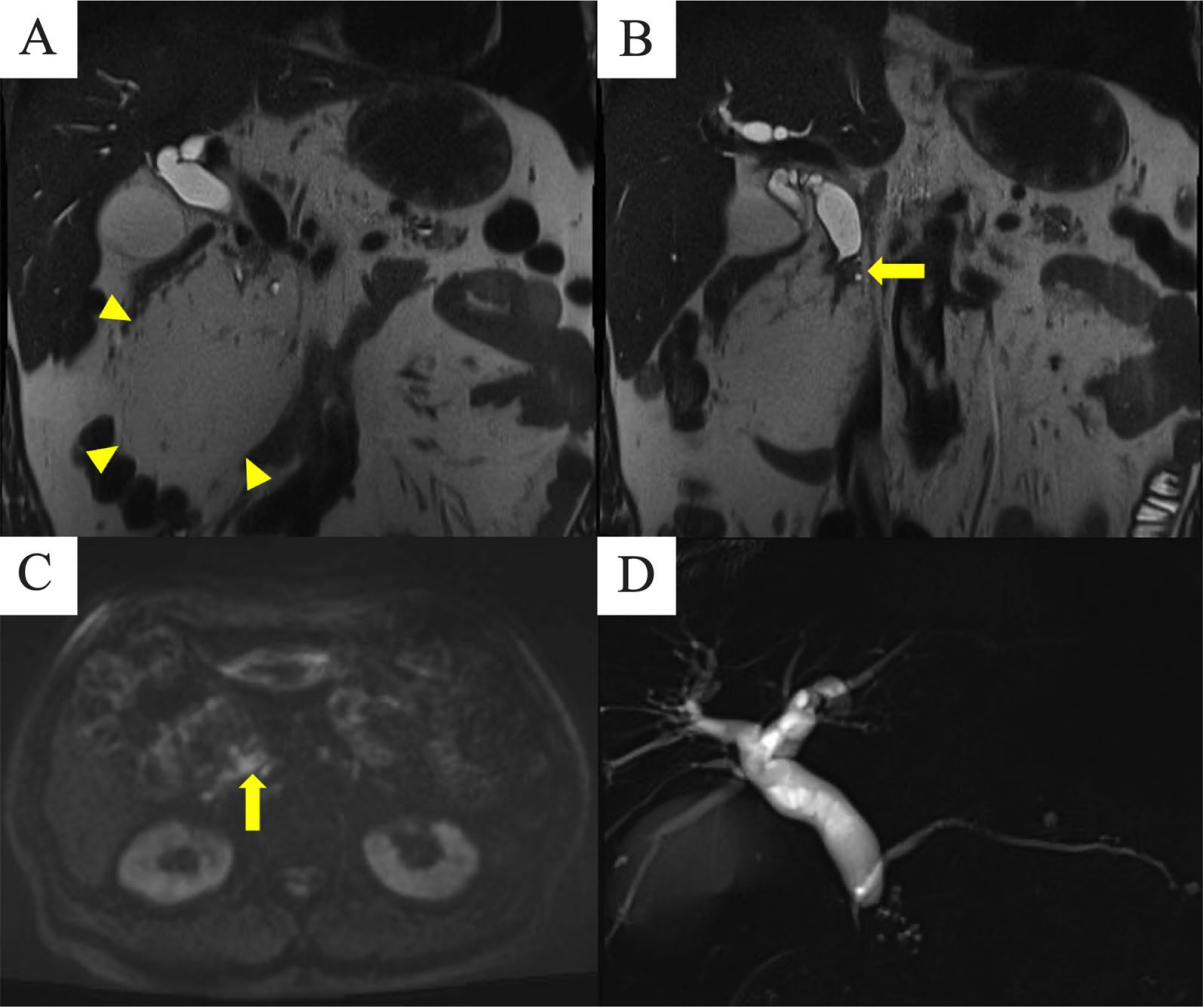
MRI before surgery. **A** The previously diagnosed 12 cm tumor with homogeneous adipose intensity is visible in the pancreatic head (arrowheads). **B** A solid T2-low intensity mass appeared at the upper margin of the tumor (arrow), which invaded and obstructed the distal common bile duct. **C** Diffusion-weighted imaging showed a high-signal intensity mass at the upper margin of the adipose tumor (arrow). **D** Magnetic resonance cholangiopancreatography showed no stenosis or other specific findings in the main pancreatic duct

During follow-up, serum analysis at his local clinic showed liver damage: aspartate aminotransferase 257 U/L, alanine aminotransferase 295 U/L, alkaline phosphatase 322 U/L, gamma-glutamyltranspeptidase, 1255 U/L; total bilirubin 1.9 mg/dL, slightly elevated inflammatory markers, white blood cell count of 4700, and C-reactive protein level was 0.3 mg/dL, and he was referred to our hospital. Serum tumor markers, including carcinoembryonic antigen, carbohydrate antigen 19–9, and DUPAN-2, were elevated (6.2 ng/ml, 81 U/ml, and 374 U/ml, respectively). Contrast-enhanced CT showed the previously diagnosed 12 cm tumor in the pancreatic head (Fig. 1C, D) and an irregular isodensity mass at the upper margin of the tumor (Fig. 1E) that invaded and obstructed the distal common bile duct. MRI showed similar findings as contrast-enhanced CT (Fig. 2A, B) and diffusion-weighted imaging (DWI) showed high signal intensity in the mass at the upper margin of the tumor (Fig. 2C). Magnetic resonance cholangiopancreatography (MRCP) revealed no stenosis or other specific findings in the main pancreatic duct (Fig. 2D). Based on these imaging findings, the patient underwent endoscopic retrograde biliary drainage and bile duct brushing cytology, which revealed indeterminate findings.

**Fig. 3:**
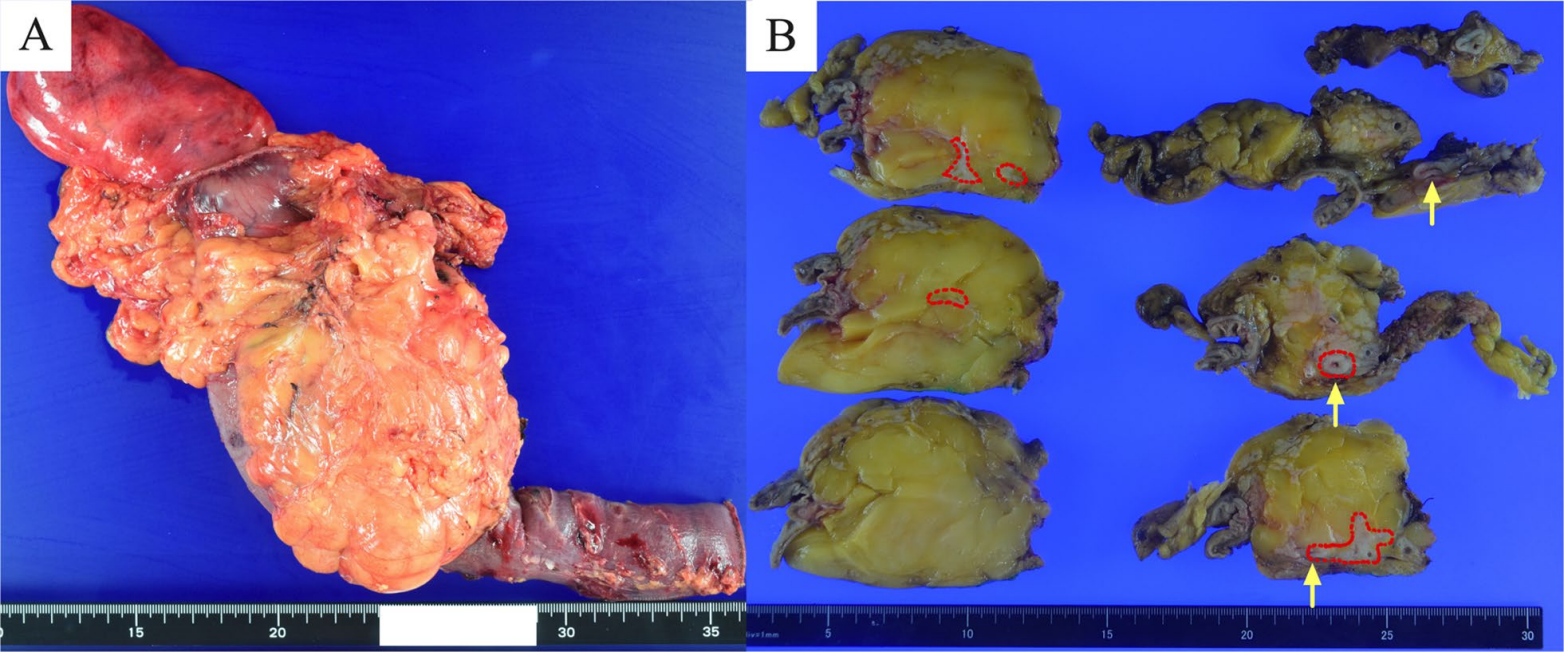
Macroscopical findings. **A** The surgical specimen of a 12 cm adipose tumor in the pancreatic head. **B** The cancerous area (red dotted line) was located around the adipose lesion and invaded the common bile duct (arrows)

**Fig. 4:**
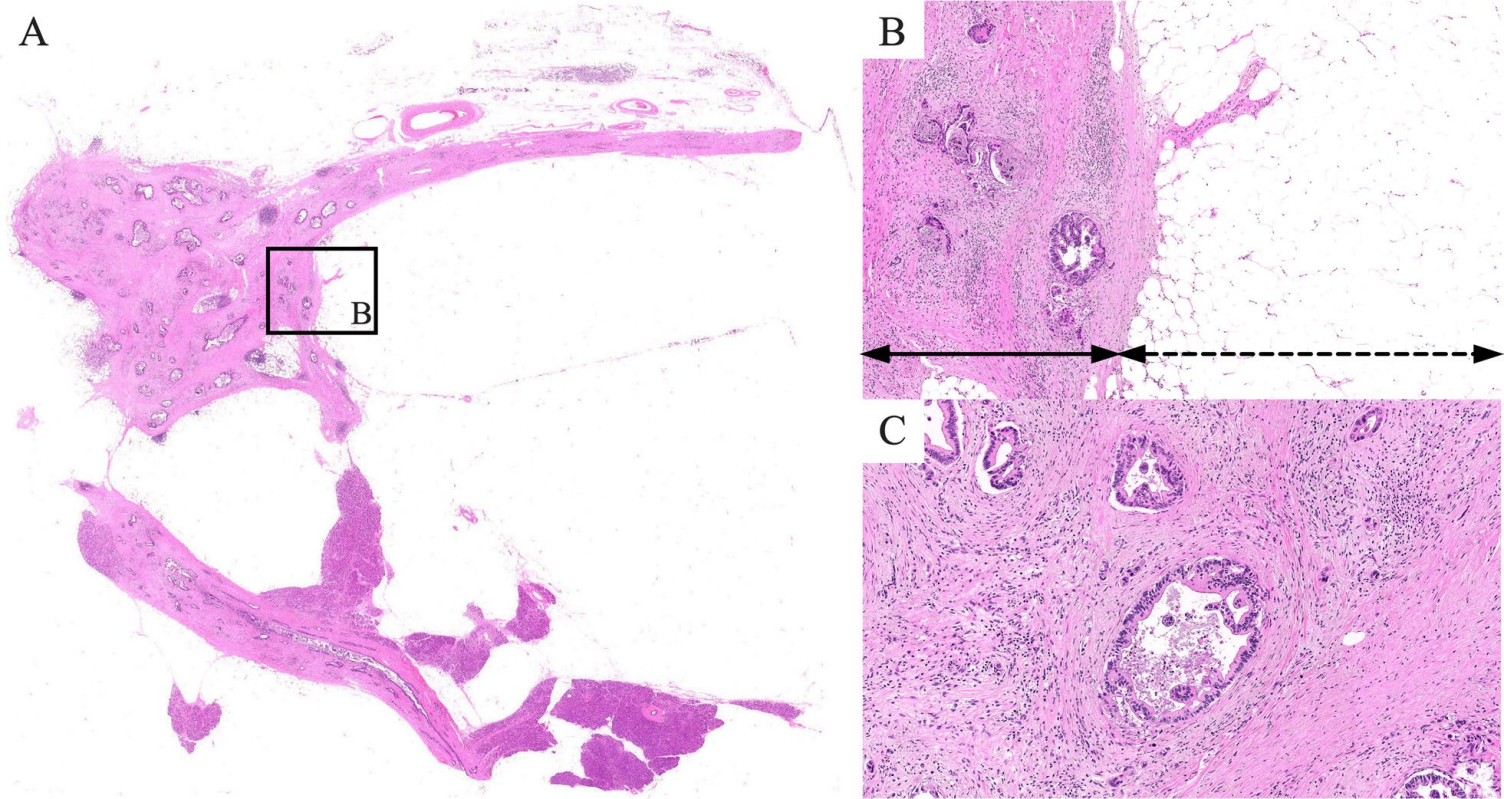
Microscopic findings. **A, B** Hematoxylin–eosin staining reveals irregularly distributed ducts with coarsely granular chromatin and enlarged nuclei (arrow) in the pancreatic parenchyma compressed by proliferating adipose tissue (dotted arrow). **C** Extensive fibrosis of the pancreatic parenchyma and atrophy of the acinar cells around the lipoma, suggesting chronic pancreatitis

The differential diagnosis of the tumor at that time was as follows: (1) pancreatic liposarcoma (focal change from well-differentiated to dedifferentiated, not a lipoma), (2) distal cholangiocarcinoma, and (3) pancreatic cancer. After the cholangitis improved, a pancreatoduodenectomy was performed. Intraoperative findings revealed a soft adipose tumor, approximately 12 cm in size, in the pancreatic head. The pancreatic parenchyma of the pancreatic body and tail was normal and soft without chronic pancreatitis and showed no abnormal findings. Histological examination of the surgical specimen showed a 12 cm adipose tumor in the pancreatic head (Fig. 3A). Figure 3B shows a whitish tumor, located around the adipose lesion and invaded the common bile duct. The largest tumor measured 35 mm in diameter. Histologically, hematoxylin and eosin staining revealed irregularly distributed ducts with coarsely granular chromatin and enlarged nuclei in the pancreatic parenchyma compressed by proliferating adipose tissue, with a diagnosis of moderately differentiated PDAC (Fig. 4A, B). Microscopically, the tumor showed lymphatic, venous, perineural, bile duct, and retroperitoneal invasions.

The adipose lesion presented homogeneous adipose tissue with no evidence of sarcoma, which led to a diagnosis of lipoma. Additionally, extensive fibrosis of the pancreatic parenchyma and atrophy of the acinar cells around the lipoma were observed, suggesting chronic pancreatitis (Fig. 4C). The resection margins were free of tumor cells, and there were no metastases to the regional lymph nodes. Taken together, the pathological diagnosis was PDAC (pT2N0M0, pStage Ib), with chronic pancreatitis and PL.

The postoperative course was uneventful, and the patient was discharged on the 15th day after surgery. The patient underwent adjuvant chemotherapy for PDAC and has remained recurrence-free for more than 6 months.

## Discussion

PLs are generally asymptomatic, rare, and benign pancreatic tumors that are commonly incidentally diagnosed based on radiological images [7]. Although surgical treatment is unnecessary in most cases, resection should be considered in cases with severe symptoms or suspected malignancy [8, 9]. In this case, during long-term follow-up for the slowly growing adipose tumor, the patient had common bile duct obstruction and the appearance of a contrast-enhanced mass at the margin of the tumor. Initially, we suspected a focal dedifferentiated change from a well-differentiated liposarcoma and performed surgery. As a result, the pathological diagnosis was PDAC coexisting with PL; to the best of our knowledge, this is the first report of a patient with this simultaneous diagnosis. The causes of this misdiagnosis are as follows: first, MRI and CT showed no abnormal findings in the main pancreatic duct; second, the patient presented with an iso-density, linear, scar-like lesion rather than a low-density solid mass typical of PDAC. Therefore, preoperative diagnosis may be difficult.

PDAC is one of the most aggressive and lethal cancers and the third leading cause of cancer mortality in the United States [10]. Despite advances in diagnostic and therapeutic techniques for several cancers, it often presents at an advanced stage, contributing to poor 5-year survival rates of 2–9% [11–13]. Therefore, a multidisciplinary management approach is recommended for the treatment of PDAC [14]. In recent years, the efficacy of neoadjuvant chemotherapy for improving the prognosis of patients with resectable PDAC has been reported [15–17]. In our case, neoadjuvant therapy might have been administered if the tumor had been preoperatively diagnosed as PDAC.

However, it was difficult to distinguish lipoma, liposarcoma, PDAC, and cholangiocarcinoma on the preoperative imaging examinations in this case. Under unusual conditions, such as the presence of a large PL, physicians should avoid stereotypes regarding imaging findings for accurate diagnosis. In addition, we did not perform endoscopic ultrasound-guided fine-needle aspiration biopsy, because needle biopsy for sarcomas is not an established approach and sometimes leads to needle tract seeding [18, 19]. Thus, we first performed surgery for diagnosis and treatment in the presented case.

Interestingly, the histopathological findings revealed chronic pancreatitis, including fibrotic and atrophic changes surrounding the PL. Previous studies have reported that chronic pancreatitis is a major risk factor for PDAC [20, 21]. The underlying mechanism of PDAC arising from chronic pancreatitis might be the local influence of the inflammatory process [22]. On the other hand, the relationship between chronic pancreatitis and PL has not been clarified. However, Jairo et al. reported that PLs compress the bile and pancreatic ducts and behave aggressively despite being benign [23].

In our case, the slow-growing large PL may have been related to chronic pancreatitis, possibly due to compression of the pancreatic parenchyma, such as obstruction of branching pancreatic ducts, or local inflammatory processes caused by adipose tissue-derived inflammatory cytokines, including adipokines. Consequently, the presence of a large lipoma may have contributed to the development of PDAC via chronic pancreatitis. However, it is difficult to test this hypothesis. Taken together, although lipomas are histologically benign, careful radiological surveillance is required for large or growing PLs.

## Conclusion

Herein, we describe an extremely rare case of PDAC arising from the pancreatic parenchyma with chronic pancreatitis compressed by a large PL. PL may be related to the development of PDAC through the surrounding inflammatory microenvironment of chronic pancreatitis. In cases of growing lipomas, careful radiologic surveillance may be needed not only for the possibility of liposarcoma but also for the coincidental occurrence of PDAC.

## Data Availability

Not applicable.

## Abbreviations

PL: Pancreatic lipoma
CT: Computed tomography
MRI: Magnetic resonance imaging
PDAC: Pancreatic ductal adenocarcinoma
DWI: Diffusion-weighted imaging
MRCP: Magnetic resonance choangiopancreatography
